# The Clinical Relevance of Tumor Biomarkers in Prostate Cancer—A Review

**DOI:** 10.3390/cancers17233742

**Published:** 2025-11-23

**Authors:** Zuzanna Majewska, Monika Zajkowska, Sara Pączek, Adam Rafał Nowiński, Weronika Sokólska, Mariusz Gryko, Karolina Orywal

**Affiliations:** 1 Department of Biochemical Diagnostics, Faculty of Pharmacy with the Division of Laboratory Medicine, Medical University of Bialystok, Waszyngtona 15A, 15-269 Bialystok, Poland; 2 Department of Neurodegeneration Diagnostics, Medical University of Bialystok, Waszyngtona 15A, 15-269 Bialystok, Poland; 3 Department of Urology, Independent Public Health Care Center of the Ministry of the Interior and Administration in Bialystok, Fabryczna 27, 15-471 Bialystok, Poland; 4 Department of Surgical Nursing, Medical University of Bialystok, 15-274 Bialystok, Poland; 5 1st Clinical Department of General and Endocrine Surgery, Medical University of Bialystok Clinical Hospital, 15-276 Bialystok, Poland

**Keywords:** prostate cancer, diagnostic biomarkers, prostate-specific antigen, PCA3, liquid biopsy, TMPRSS2-ERG, prolaris, decipher, ConfirmMDx

## Abstract

Prostate cancer is one of the most common malignancies in men worldwide. Therefore, there is an urgent need to develop accurate, accessible biomarkers for diagnosis, prognosis, and therapy monitoring. Tumor markers, which can be measured in blood, urine, or tissue, provide valuable information regarding tumor presence, progression, and response to treatment. This review provides a comprehensive overview of routinely used diagnostic and emerging biomarkers for prostate cancer, based on a systematic MEDLINE/PubMed search. Key biomarkers analyzed include PSA and its derivatives, PCA3, and TMPRSS2-ERG, as well as genomic tests such as Prolaris, Decipher, and ConfirmMDx, and liquid biopsy-based tests such as ExoDx Prostate and SelectMDx. This narrative review demonstrates that although PSA remains the mainstay of prostate cancer diagnosis, emerging molecular and genomic biomarkers are enhancing diagnostic specificity, refining risk stratification, and enabling more personalized patient care. The integration of routinely used and novel biomarkers can improve early detection, optimize treatment decisions, and ultimately improve outcomes of prostate cancer patients.

## Simple Summary

Prostate cancer is one of the most common cancers in men, underscoring the importance of accurate and accessible biomarkers for diagnosis, predicting outcomes, and therapy monitoring. This review summarizes the biomarkers currently in use and those that are emerging, as identified through a systematic MEDLINE/PubMed search. While commonly used markers such as PSA and its derivatives remain central to diagnosis, newer biomarkers—including PCA3, TMPRSS2-ERG, and genomic tests such as Prolaris, Decipher, and ConfirmMDx—offer greater specificity and prognostic value. Liquid biopsy-based assays, such as ExoDx Prostate and SelectMDx, further enhance the range of non-invasive testing options available. Overall, integrating traditional and novel molecular biomarkers enhances early detection of prostate cancer, enables more precise risk stratification and supports personalized treatment strategies, leading to improved patient outcomes.

## 1. Introduction

Prostate cancer (PCa) is the most common malignancy in the world’s male population and the second leading cause of cancer-related mortality after lung cancer [1]. More than 95% of prostate cancer cases are adenocarcinomas, with nearly 80% originating from the luminal or the basal epithelial cells in the peripheral regions, which accounts for over 70% of the total prostate gland [2].

Numerous studies have demonstrated that genetic factors play a significant role in the etiology of PCa. PCa is considered an inherited malignancy, and genome-wide analyses have identified multiple susceptibility loci associated with increased disease risk. One example is the single-nucleotide polymorphism (SNP) rs339331, which enhances the expression of the oncogenic RFX6 gene through a functional interaction with the PCa susceptibility gene HOXB13 [3]. In addition to HOXB13, other genes implicated in the heritability of PCa are HPC1 (1q24-25), PCAP (1q42-43), HPCX (Xq27-28), CAPB (1q36), and HPC20 (20q13) [4]. Additionally, numerous susceptibility mutations have been identified in DNA repair genes, including BRCA2, ATM, CHEK2, BRCA1, RAD51D, and PALB2 [5]. In addition, there is ethnic variation in the incidence of cancer, with the highest rates of incidence and mortality in African men and the lowest rates in Asian men [6]. Moreover, elevated body mass index (BMI) also strongly correlates with prostate cancer mortality and is considered as a risk factor for the progression of low-grade to high-grade prostate cancer [7]. PCa predominantly affects older men, with 6 out of 10 cases being diagnosed in those aged 65 years or older, and the average age at diagnosis being approximately 66 years. Statistical models indicate that 1 in 7 men will be diagnosed with PCa and that 1 in 39 men will die of the disease, while the remaining cases will be affected by a slow-growing tumor or live because of effective treatment [8]. Many prostate cancers progress slowly and are limited to the prostate gland, requiring minimal or, in some cases, no treatment. However, other forms are aggressive, capable of rapid spread, and often remain largely incurable despite intensive multimodal therapy. Metastatic disease is the principal cause of prostate cancer-related mortality. Patients die because of the prostate cancer mostly due to metastases to the pelvic and retroperitoneal lymph nodes, spinal, bladder, rectum, bone, and brain [9]. Localized prostate cancer is asymptomatic. When symptoms become present, the disease is generally too advanced for cure.

**Figure 1:**
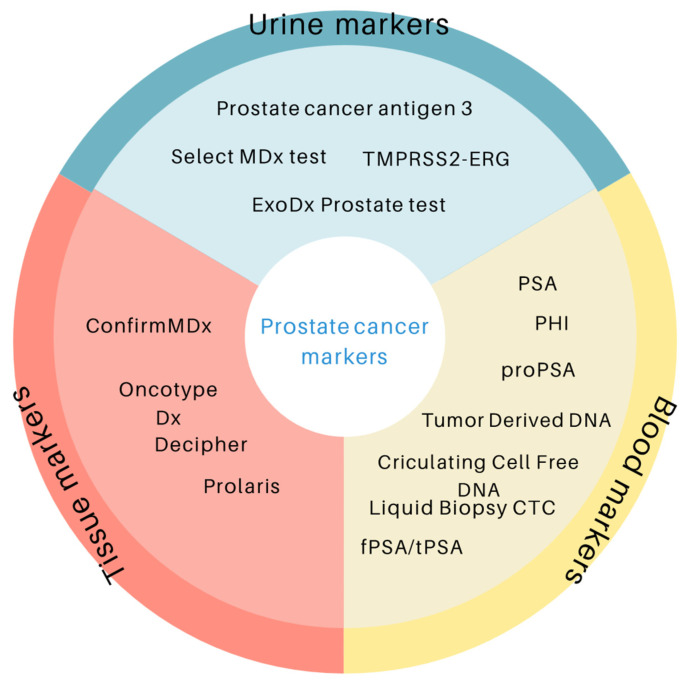
Prostate cancer biomarkers depending on the type of material tested.

As the incidence of malignant neoplasms continues to rise, the issue of diagnostics, increasing the chances of early cancer detection, is extremely important. Currently, prostate cancer diagnoses rely on digital rectal examination, prostate-specific antigen determination, and prostate biopsies. Improved blood, urine markers and tissue markers, used alone or in combination, are needed to enhance diagnosis, differentiation, prognosis, and therapy monitoring (Figure 1). Recent advances in newly developed research methodologies have led to significant progress in research based on identification of prostate cancer biomarkers, supporting the emergence of diagnostics, prognostic, and predictive markers, which are discussed in this article.

## 2. Methods

**Figure 2:**
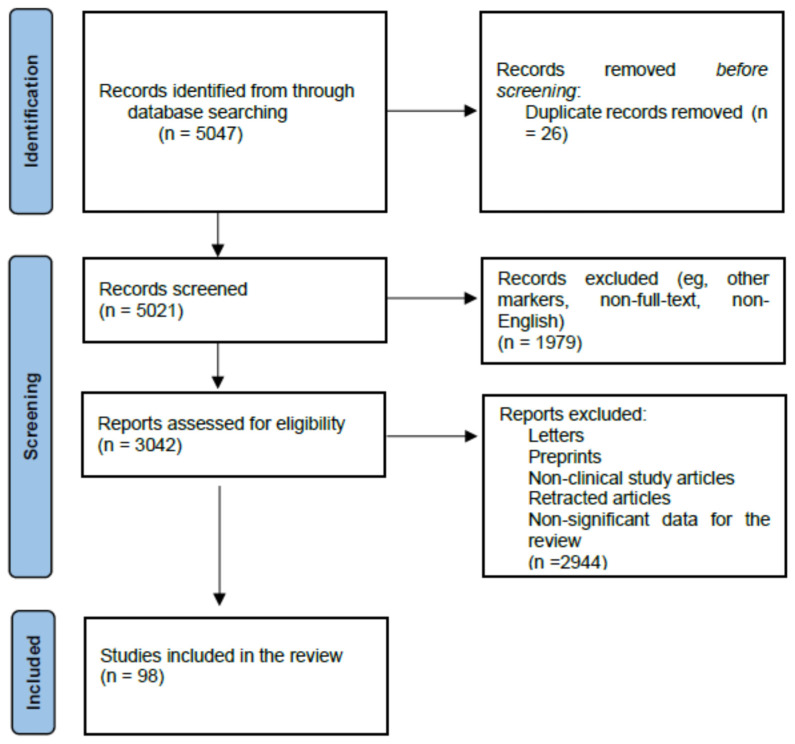
Prisma 2020 flow diagram depicting the methods for including studies in the review.

A comprehensive literature search was conducted in the MEDLINE/PubMed electronic database covering the past five years (from 1 July 2020 to 30 June 2025), using the keyword: “prostate cancer markers” (*n* = 3311). In the next step, specific searches were performed to identify key prostate cancer biomarkers relevant to this review, including: “PSA and its derivatives and prostate cancer” (*n* = 569), “liquid biopsy and prostate cancer” (*n* = 546), “prostate cancer antygen 3 and prostate cancer” (*n* = 54), “select MDx test and prostate cancer” (*n* = 5), “TMPRSS2-ERG and prostate cancer” (*n* = 176), “ExoDx Prostate test” (*n* = 26), “confirm MDX and prostate cancer” (*n* = 7), “prolaris and prostate cancer” (*n* = 38) and “decipher and prostate cancer” (*n* = 315). Subsequently, only English-language, full-text publications limited to studies involving human subjects were included. Finally, letters, non-clinical studies, retracted articles, and papers presenting non-relevant data were excluded from the analysis (Figure 2).

A formal quality assessment of the included studies was not performed as the objective of this review was to provide a narrative synthesis of current clinical applications and emerging trends in prostate cancer biomarker research rather than a systematic meta-analysis. The absence of a formal quality appraisal is acknowledged as a limitation of this study.

## 3. Prostate Cancer Markers

**Figure 3:**
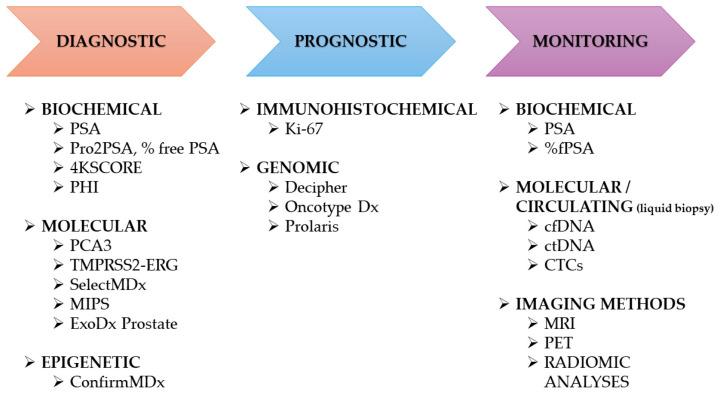
Prostate cancer biomarkers depending on their application.

Disease-related biomarkers can serve as valuable adjuncts in cancer diagnostics. Screening provides an opportunity to detect cancer at an early stage, before the onset of symptoms and metastasis (Figure 3). Early detection of cancer can reduce morbidity and improve patient survival.

### 3.1. Blood Markers

#### 3.1.1. PSA and Its Derivatives

PCa incidence has risen, largely due to the widespread use of prostate-specific antigen (PSA), which was determined in 1986 [10]. PSA is a kallikrein-like serine protease produced and secreted by the epithelial cells of the prostate gland. Common methods for detecting PCa include measuring serum PSA concentration, digital rectal examination, and transrectal ultrasonography. Most of the PSA in the serum is bound to proteins, mostly α-antichymotrypsin, constituting the so-called complexed PSA (cPSA): the unbound part is referred to as free PSA (fPSA). Elevated PSA level can reflect the presence of cancer, but can also be caused by benign prostatic hyperplasia (BPH), prostatitis, ejaculation, or exercise (false positive). Although the threshold of the serum tPSA is 4 ng/mL, the patient’s age should be taken into account. For men under 50, a lower cut-off value of 2.5 ng/mL is recommended to improve early detection [11]. It has been reported that the specificity of PSA alone is quite low when the mentioned cut-off values are used, leading to both false negative and false positive results [12]. Low specificity and limited accuracy of PSA as a biomarker make it difficult to distinguish between indolent (slow-growing) and aggressive cancer. As a result, many men undergo unnecessary biopsies and overtreatment, which can cause physical and psychological harm. At the same time, some men with aggressive PCa are missed, highlighting the need for more precise diagnostic tools. It should be emphasized that prostate biopsies are associated with certain potential complications, including bleeding, urinary retention, or even infection. Consequently, substantial efforts have been directed toward developing novel strategies to enhance the specificity of PSA testing for PCa detection. Among these approaches are dynamic indicators that reflect temporal changes in PSA concentration, such as PSA velocity (PSAV) and PSA doubling time (PSADT). Although their diagnostic utility remains limited, these parameters may serve as valuable prognostic biomarkers in patients undergoing post-treatment surveillance for prostate cancer [13]. However, PSA density (PSAD), defined as ratio of serum PSA concentration to the volume of the prostate gland, has demonstrated certain advantages over PSA in the detection of PCa, particularly identifying the most aggressive forms [14]. Recent studies has also shown that integrating PSAD and MRI findings enables a more individualized approach to prostate biopsy strategies that is acceptable to both patients and clinicians seeking to minimize unnecessary procedures [15].

The free-to-total serum PSA ratio, typically expressed as a percentage (%fPSA), has been proposed as a tool to distinguish benign from malignant prostate conditions in men with normal digital rectal examination findings and total serum PSA levels within the 4–10 ng/mL range. Although no definitive %fPSA has been universally established, lower ratios are generally associated with an increased likelihood of PCa in patients with PSA values below 10 ng/mL [16]. Evidence suggest that a f/tPSA ratio lower than 0.1 in individuals within the so-called “grey zone” of PSA concentrations is associated with malignancy in only approximately 42% of cases, leading to a considerable number of unnecessary biopsies [17].

In an effort to enhance the limited diagnostic performance of PSA-based testing, several studies have investigated pro-PSA (also referred to as [−7]pro-PSA), which is known as truncated isoform of PSA containing a seven-amino acid N-terminal pro-leader peptide. Pro-PSA is undergoes partial cleavage by members of the human kallikrein family and other proteases, leading to the formation of three truncated derivatives: [−5]pro-PSA, [−4]pro-PSA, and [−2]pro-PSA. Among these, [−2]pro-PSA production is selectively upregulated in malignant tissue and shows a significant association with high-grade PCa [18]. There are also studies indicating greater diagnostic usefulness of calculating the ratio of proPSA to fPSA in the detection of prostate cancer in men with a negative digital rectal examination result and a tPSA concentration of 2–10 ng/mL [19].

Prostate health index (PHI) is a multifactorial mathematical model that combines serum concentrations of three PSA isoforms: total PSA, %fPSA, and [−2]proPSA. This composite index has been developed to enhance the accuracy of PCa detection. In a multicenter prospective study involving 658 men, aged 50 years and older, with total PSA levels between 4 and 10 ng/mL and normal digital rectal examination, PHI demonstrated superior diagnostic performance compared with its individual components—total PSA, %free PSA and [−2]proPSA—in identifying clinically significant PCa, including cases with a Gleason score of 7 or higher. PHI achieved the highest AUC for overall PCa (AUC phi 0.708), outperforming %fPSA (AUCs 0.648), [−2]proPSA (AUCs 0.550), and total PSA (AUCs 0.516). Moreover, the authors revealed that at sensitivity threshold of 90% for detecting significant PCa, the use of PHI could have potentially avoided unnecessary prostate biopsy in approximately 30.1% [20]. Therefore, PHI was approved by the FDA (Food and Drug Administration) and has been established as a PCa biomarker for men over 50 years of age with negative digital rectal examination and PSA in the grey zone (between 4 and 10 ng/mL) to improve diagnostic accuracy and avoid unnecessary biopsies (Table 1). PHI also correlates with tumor volume and can predict pathological outcomes and tumor recurrence in radical prostatectomy patients [21,22].

In recent years, PHI density (PHID: PHI/prostate volume) has gained increasing attention for its clinical utility in PCa diagnosis. Several studies have demonstrated that PHID provides higher diagnostic accuracy for PCa detection, with an AUC of 0.84, outperforming other PSA-derived parameters. Furthermore, it has been reported that PHID could potentially reduce unnecessary biopsies by approximately 38%, while missing only about 2% of cancer cases [28]. Beyond diagnostic performance, PHID has also been shown to correlate with cancer aggressiveness, especially in post-radical prostatectomy cases characterized by high-grade tumors or extracapsular prostatic invasion. Incorporating PHID into preoperative evaluation may assist clinicians in patient counseling, aid in selecting candidates suitable for active surveillance, and support individualized treatment decision-making [22].

The 4Kscore test integrates clinical parameters—such as patient age, abnormal digital rectal examinations, and prior prostate biopsy history—with the serum concentrations of total PSA, free PSA, intact PSA, and human kallikrein 2 (hK2). Human kallikrein-related peptidase 2 is a secreted serine protease, which shares approximately 80% sequence homology with PSA and is also predominantly expressed in the prostate gland, both benign and malignantly tissue. However, its enzymatic activity differs from that of PSA. This test has demonstrated clinical utility in the prediction of prostate cancer biopsy outcomes in men with elevated PSA levels, abnormal DRE results or after a prior negative biopsy, but persistently abnormal PSA levels. Importantly, it significantly reduces the number of unnecessary prostate biopsies [29]. Comparative analyses have shown that PHI and the 4K panel had a similar, high diagnostic accuracy for detecting overall and high-grade PCa, with favorable sensitivity and specificity profiles. Therefore, both biomarkers can aid in minimizing the number of unnecessary prostate biopsies. However, the 4K score test has not yet received FDA approval [30].

#### 3.1.2. Liquid Biopsy (Circulating Tumor Cell (CTC), Tumor Derived (ct) DNA, Circulating Cell Free (cf) DNA)

Recent advances in microarray technologies and next-generation sequencing approaches have enabled detailed characterization of aberrant epigenetic patterns in the genomes of prostate cancer cells. The application of analyses based on circulating cancer cells or their molecular products obtained from blood or other body fluids (commonly referred to as liquid biopsy) offers broad opportunities for the early detection of cancer and its recurrence, with individual risk assessment and therapy monitoring. It also enables a comprehensive view of the entire disease, as cancer cells or their molecular markers are released from both primary tumors and metastatic sites. This provides insight into tumor evolution, the identification of novel therapeutic targets, and the elucidation of mechanisms underlying therapy resistance [31]. CTCs (Circulating Tumor Cells) and ctDNA (tumor-derived DNA) are among the most prominent liquid biopsy markers detectable in the blood and bone marrow of patients with prostate cancer. Quantification of CTCs and enables noninvasive and serial monitoring of tumor burden and tumor biology in both pre- and post-treatment scenarios, and also provides prognostic value for patient survival [32]. In prostate cancer, there are both primary CTCs, which are detected at the diagnostic stage, and secondary CTCs, which are found after radical treatment. Studies have shown that the absence of primary CTCs in the blood of prostate cancer patients is associated with a lower risk of micrometastases. The development of bone metastases involves CTCs, and therefore their presence in the bloodstream correlates with the presence of metastases [33]. The most validated and currently only FDA-approved commercially available immunoaffinity platform for identifying CTCs is the CellSearchTM system (Janssen Diagnostics, LLC, Raritan, NJ, USA), and this test is an independent predictor of overall and progression-free survival in metastatic prostate cancer, better than all PSA-based algorithms [34]. A complementary approach to analyzing CTCs is the identification of small nucleic acid fragments released from tumor cells—cell-free ctDNAs and tumor-derived DNA. Studies have shown that seminal plasma contains higher cfDNA (cell-free DNA) concentrations than blood plasma, likely due to its proximity to the prostate tumor region. cfDNA consists of both single-stranded (ssDNA) and double-stranded DNA (dsDNA); therefore, the best method is to determine the total cfDNA, which gives the best information about the mass of the tumor and has enabled tracking of different emerging genetic aberrations present in the tumor in “real-time” [35]. Assessment of circulating tumor DNA fraction represents a valid and robust biomarker, particularly in castration-resistant prostate cancer (CRPC), and can predict clinical outcomes as well as monitor disease evolution during treatment. Moreover, higher CTCs counts are consistently associated with poorer overall survival in patients with this malignancy [36]. Determining the CTCs gene expression profile for cancer in a given patient helps to apply an personalized treatment regimen, and evaluate therapy effectiveness and help identify mechanisms of drug resistance.

### 3.2. Urine Markers

#### 3.2.1. Prostate Cancer Antigen 3 (PCA3)

In addition to PHI, which is analyzed in blood samples, the U.S. FDA has approved the PCA3 assay (Progensa PCA3, Hologic Inc., Marlborough, MA, USA) as a molecular marker measured in urine, though its use in currently limited to the repeat biopsy setting. PCA3 is a non-coding RNA highly specific to the prostate gland and markedly overexpressed in prostate cancer cells. Notably, its expression level is independent of total PSA, patients age, prostate volume, and inflammatory status [37]. The PCA3 assay can be particularly useful in guiding decisions regarding repeat biopsy in men with one or more prior negative prostate biopsies. This test quantifies the prostate cancer gene 3 (PCA3) and PSA RNA levels in post-DRE first-catch urine specimens to aid in the detection of prostate cancer among men aged 50 years or older with elevated serum PSA and history of negative biopsy results [38]. The test result is reported as the ratio of PCA3 RNA to PSA RNA, called the “PCA3 score”, where a score below 25% is negative and associated with a lower likelihood of positive biopsy [39]. It was shown that PCA3 test results of 35 showed diagnostic sensitivity ranging from 58% to 82%, diagnostic specificity from 58% to 76%, positive predictive value (PPV) ranging from 67% to 69%, negative predictive value (NPV) from 87% and area under the curve (AUC) from 0.68 to 0.87 [40,41]. The recent study of Solemani et al. found that higher urinary PCA3 levels correlate with the presence of unfavorable pathological features of prostate cancer (in addition to perirectal invasion) in patients undergoing radical prostatectomy. The results suggest that PCA3 may be a useful marker to support therapeutic decisions, especially in patients with intermediate- and high-risk of prostate cancer [42]. Some studies have shown that PCA3 also correlates with tumor volume and can predict non-significant tumors (TV < 0.5 mL), but its value in predicting tumor aggressiveness (Gleason score ≥ 7, ECE) remains limited and inconclusive. In addition, the PCA3 assay has also been shown to lack utility as a marker for monitoring tumor progression, as does not correlate clearly with progression parameters [43]. The PCA3 test has also been included in the European Association of Urology (EAU) and NCCN 2020 guidelines for decision-making about potential re-biopsy. The important fact is that PCA3 seems to be more specific for prostate cancer and not elevated due to benign prostatic hyperplasia (BPH)—unlike prostate-specific antigen (PSA) or other non-cancerous conditions [44].

#### 3.2.2. Select MDx Test

The SelectMDx test is a non-invasive diagnostic test for prostate cancer. It is performed in urine after a prostate massage to assess the risk of clinically significant prostate cancer (csPCa). It is particularly useful when deciding whether to perform a prostate biopsy or not. This test measures the mRNA levels of three cancer-related genes: DLX1 (a prostate cancer cell proliferation gene), HOXC6 (a prostate cancer progression gene), and KLK3 (a prostate cancer reference gene). The expression of above-mentioned genes is associated with clinical risk factors for prostate cancer (PSA level, prostate volume, age, family history, digital rectal examination (DRE) findings, etc.) [45]. In the van Neste et al. study, a clinically non-invasive test based on mRNA analysis of urinary biomarkers HOXC6 and DLX1 was developed and validated for the detection of clinically significant prostate cancer (Gleason ≥ 7). The integration of these biomarkers with traditional risk factors (such as PSAD and biopsy history) in a logistic regression model achieved high diagnostic accuracy (AUC up to 0.90), outperforming previously used tools such as the PCPTRC calculator and PCA3 test. The developed model allows for improved risk stratification and a reduction in the number of unnecessary biopsies, underscoring its value in the context of the clinical use of tumor markers in prostate cancer diagnosis [46]. Select MDx provides a risk assessment indicating the likelihood of high-grade prostate cancer (Gleason score ≥ 7). A key advantage of this test is its ability to significantly reduce the number of unnecessary biopsies, thereby helping to avoid invasive procedures in men classified as low risk [47]. The test is recommended for men who have an elevated PSA level, but the diagnosis has not been confirmed, as well as for individuals with a family history of prostate cancer. In contrast, the study of Lendínez-Cano et al. confirmed the moderate performance of the SelectMDx test in detecting clinically significant prostate cancer (ISUP > 1) in patients with PSA levels of 3–10 ng/mL and normal DRE results. Although the test yielded worse results than previously reported (AUC 0.63), its performance was comparable to that of the ERSPC+DRE risk calculator and MRI. Combining SelectMDx with MRI improved the negative predictive value (NPV) to up to 93% and may be a useful tool for further diagnosis; however, additional studies are needed to assess its cost-effectiveness [48].

#### 3.2.3. TMPRSS2-ERG

TMPRSS2-ERG (T2E) is a gene fusion present in approximately 50% of prostate cancer cases and represents one of the most common genetic alterations observed in patients with this type of cancer [49]. It arises as a result of a fusion between the TMPRSS2 gene (transmembrane protease, serine 2) and the ERG transcription factor (ETS-related gene). This rearrangement results in ERG overexpression, which contributes to prostate cancer development [50]. The material for testing the TMPRSS2-ERG gene fusion is urine obtained after rectal prostate massage and tissue samples. A positive TMPRSS2-ERG test result in urine suggests the presence of prostate cancer, although it does not necessarily indicate its aggressive nature [50]. A study by Yi et al. demonstrated the presence of the TMPRSS2:ERG gene fusion in 45.16% of patients with prostate cancer, while it was not found in patients with benign prostatic hyperplasia or in cell lines. Although no significant differences were observed in the total Gleason score, patients with the fusion exhibited a higher Gleason score within the dominant component. These findings suggest that the presence of TMPRSS2:ERG fusion is associated with a higher degree of prostate malignancy [51]. Furthermore, the presence of this fusion found in circulating tumor cells (CTCs) may indicate a high metastatic potential. TMPRSS2 is regulated by androgens, which have been shown to influence the favorable tumor response to androgen deprivation therapy (ADT) [52]. Currently, numerous studies are underway on targeted therapies that specifically block ERG-related oncogenic pathways [53,54,55].

#### 3.2.4. ExoDx Prostate Test

The ExoDx Prostate Test (EPI) is a non-invasive, urine-based biomarker assay that is designed to assess the risk of developing clinically significant prostate cancer (Gleason score of at least 7). Unlike PSA testing, the EPI is not influenced by prostate size or inflammation, and a digital rectal examination is not required before urine collection [56]. This improves patient comfort and sample standardization. The test measures the expression levels of three exosomal RNA biomarkers—ERG (ETS-related gene), PCA3 (prostate cancer antigen 3), and SPDEF (SAM pointed domain-containing ETS transcription factor)—which are associated with prostate tumor formation [57]. The EPI is particularly useful for men with intermediate PSA concentrations (2–10 ng/mL), for whom a biopsy decision is uncertain, as well as for men aged 50 years or older and for those with a previously negative biopsy but persistent risk [58,59]. By analyzing gene expression within urinary exosomes—small extracellular vesicles (30–200 nm) encapsulated by a lipid bilayer that protects RNA, DNA, and proteins—the test enables accurate molecular profiling of tumor-derived material from biofluids without the need for direct tissue sampling [60]. The exosomal microenvironment preserves RNA integrity, enabling precise RNA expression analysis. Multiple prospective clinical studies have validated the EPI assay for predicting high-grade prostate cancer (≥Grade Group 2), demonstrating its ability to identify clinically significant disease while reducing unnecessary biopsies in men at low risk [61].

### 3.3. Tissue Markers

#### 3.3.1. ConfirmMDx

In contrast to the previously discussed markers, the ConfirmMDx assay requires an invasive diagnostic procedure—a prostate biopsy. This method enables the assessment of the methylation status of genes commonly present in PCa. According to the literature, the following genes are taken under analysis: Adenomatous Polyposis Coli (APC), Glutathione S-S-Transferase Pi 1 (GSTP1), and Ras association domain family member 1 (RASSF1) [62]. Current evidence indicates that a cancer-negative biopsy accompanied by a positive ConfirmMDx test suggest the presence of latent prostate cancer that may have been missed in prior evaluations. Therefore, the National Comprehensive Cancer Network guidelines recommend this test for men with an elevated PSA blood level and a prior negative prostate biopsy [63]. In addition, results obtained from multiple studies confirmed the enhanced negative predictive value when using this test compared to histopathologic review alone [64,65,66,67]. It is also worth noting that the implementation of this assay has been associated with a significant reduction in the number of repeat biopsies performed in patients undergoing evaluation for prostate cancer [66]. The application of this assay is mainly used to personalize treatment for men with low- or intermediate-risk- risk prostate cancer (I-PCa) [65]. Similarly to the ConfirmMDx, the GPS test relies on fixed biopsy material obtained from a patient. The principle of this assay is based on the quantitative measurement of the expression of 17 genes in mRNA using the enzyme reverse transcriptase in conjunction with polymerase chain reaction (RT- PCR). The test generates a score on a 0–100 scale, which reflects the aggressiveness of the tumor and enables the prediction of adverse for pathological features (Gleason = 4 + 3 and/or pT3+) [66,67]. According to research, this assay may also constitute a significant predictor of biochemical recurrence and metastatic progression [68]. Several limitations of the OncotypeDx test have been documented in the literature, including the lack of prospective validation for its clinical utility in randomized clinical trials [69]. In addition, compared with standard blood-based biomarkers, tissue-based gene expression signatures remain expensive. Finally, tissue heterogeneity in prostate cancer may result in variable outcomes depending on the biopsy location [70,71]. Despite the aforementioned restrictions, current NCCN guidelines indicate that the OncotypeDx Genomic Prostate Score, as well as Decipher and Prolaris, may be employed to stratify risk in men with low- to intermediate-risk prostate cancer [72].

#### 3.3.2. Prolaris

Prolaris (CCP) is a genomic assay designed to evaluate the cancer cell cycle progression in patients with low-and intermediate-risk-prostate cancer. In practice, this test estimates the expression of multiple genes in tumor tissue and assess the rate of cell proliferation [73]. The score-based result is derived from the analysis of expression changes in 31 cell cycle progression genes and 15 housekeeping genes. The score ranges from 0 to 10; each one-unit increase represents a twofold enhancement in risk of disease development [74]. Additionally, the CCP report also provides an individualized estimate of a patient’s 10-year risk of prostate cancer-specific mortality. The analytical validity and reliability of the Prolaris assay have been proven in both biopsy and radical prostatectomy (RP) specimens [74]. Therefore, the potential clinical utility of the CCP assay lies in its ability to refine individual patients’ risk assessment. However, literature states there is not enough evidence proving that the Prolaris assay to significantly influences patient clinical outcomes. On the contrary, the results of multiple studies suggest that performing this assay does not have an essential impact on the strategy of treatment, while being associated with increased costs in prostate cancer diagnosis [74].

#### 3.3.3. Decipher

According to published reports, this genomic assay demonstrates the broadest application in PCa diagnostics and has been approved by the Centers of Medicare and Medicaid Services [75]. Decipher assesses the expression of 22 genes identified through a whole-transcriptome microarray analysis. This analysis was originally developed to support the prognostication in patients who have undergone radical prostatectomy following the diagnosis of various prostate cancer phenotypes. For this purpose, it utilizes an algorithm based on the expression of 22 RNA biomarkers linked to androgen receptor signaling, cell differentiation and proliferation, as well as motility and immune modulation [76]. In 2023, a study conducted by Benjamin Behers et al. was published to evaluate the correlation between Decipher assay results and the actual clinical outcomes of patients who underwent robotic prostatectomy over an 8-year period. The findings suggest a negative relationship between the Decipher prediction of Genomic Gleason Score of Primary 4 or 5 and no disease progression. Higher Post-Operative Radiation Response scores were found to be associated with increased mortality overall [77]. Additional studies provide consistent evidence that the aforementioned test helps in determining tumor aggressiveness, which in turn facilitates more personalized treatment decision-making (Figure 3) [78]. In addition, a study involving 61 patients with persistent prostate-specific antigen (pPSA) showed that, in a multivariate analysis, only Decipher Score and preoperative PSA levels were significant predictors of persistent pPSA [78]. Although this genomic test can provide valuable information regarding tumor malignancy, thereby enabling treatment stratification for patients diagnosed with prostate cancer, particularly those with low- or intermediate-risk, current guidelines from prominent medical organizations, such as the American Society for Clinical Oncology, do not recommend its use in routine diagnostics [79,80].

## 4. Conclusions

In conclusion, our review highlights the significant progress that has been made in the field of prostate cancer biomarkers (Table 2).

While traditional markers, including PSA and its derivatives, continue to play a crucial role in diagnosis and treatment monitoring, new molecular and genetic tests, including PCA3, TMPRSS2-ERG, and genomic classifiers such as Prolaris and Decipher, offer enhanced specificity and risk stratification (Table 3). Moreover, liquid biopsy methods and novel diagnostic tests, such as the ExoDx Prostate and SelectMDx assays, hold the potential to reduce the number of unnecessary biopsies (Table 2).

Looking to the future, research should focus on standardizing biomarker assessment methods, multicentre validating combined biomarker panels, and integrating molecular, genomic and imaging (radiomic) data into clinically applicable diagnostic algorithms. Incorporating emerging technologies, such as circulating tumor DNA and multi-omic profiling, could improve the precision of disease detection, prognosis, and therapy selection, and contribute to a more personalized management of prostate cancer patients. Recent advances have also emphasized the increasing importance of combining molecular and imaging-based information to improve diagnostic accuracy and treatment stratification. Multiparametric MRI (mpMRI), PET imaging and radiomic analyses offer additional insights into tumor heterogeneity and the tumor microenvironment, thereby enhancing the predictive value of existing biomarkers [98]. Developing integrative, multi-omic diagnostic frameworks that combine genomic, transcriptomic and imaging data is a promising approach for the next generation of precision oncology in prostate cancer.

## Figures and Tables

**Table 1:** List of FDA-approved prostate markers.

Test	Sample Type	Clinical Indication	Year of FDA Approval	References
Total PSA	Blood	Screening and diagnosis	1986	[23]
Free PSA	Blood	Screening and diagnosis	1997	[23]
CellSearchTM system	Blood	Monitoring patients with metastatic prostate cancer	2004	[24]
Pro2PSA	Blood	Screening and diagnosis	2012	[23]
PHI	Blood	Diagnostic accuracy improvement	2012	[25]
PCA3	Urine	Re-biopsy decision	2012	[26]
4Kscore	Blood	Help in deciding on a prostate biopsy	2021	[27]

**Table 2:** Prostate cancer biomarkers.

Biomarker	Sample Type	Mechanism	References
PSA and derivatives (f/t PSA, PHI, PHID, 4Kscore)	Serum	Kallikrein-related proteases	[10,11,12,13,14,15,16,17,18,19,20,21,22,23,24,25,26]
Liquid biopsy (CTC, ctDNA, cfDNA)	Blood	Circulating tumor material	[22,27,28,29,30,31]
PCA3	Urine	Non-coding RNA	[32,33,34,35,36,37,38,39]
SelectMDx	Urine	DLX1, HOXC6 mRNA	[40,41,42,43]
TMPRSS2-ERG	Urine/tissue	Gene fusion	[44,45,46,47,48,49,50]
ConfirmMDx	Tissue	Gene methylation (APC, GSTP1, RASSF1)	[51,52,53,54,55,56]
OncotypeDx GPS	Tissue	17-gene RT-PCR panel	[57,58,59,60,61,62,63,64,65,66,67]
Prolaris (CCP)	Tissue	31 cell cycle genes	[68,69]
Decipher	Tissue	22 RNA biomarkers	[70,71,72,73,74,75]

**Table 3:** Comparison of diagnostic indicators of prostate cancer biomarkers.

Biomarker	Sensitivity	Specificity	AUC	Ref.
PSA	~72–86%	~30–47%	0.55–0.70	[81,82]
PHI (Prostate Health Index)	~90%	~31–66%	0.70–0.78	[83,84]
4Kscore	~86–97%	~61–72%	0.82–0.90	[85,86]
PCA3	~55–69%	~74–83%	0.66–0.75	[87,88]
TMPRSS2-ERG fusion	~24–93%	~93–97%	0.63–0.74	[89,90]
SelectMDx	~76–91%	~49–73%	0.76–0.87	[91,92]
ExoDx Prostate (EPI)	~92%	~34–47%	0.70–0.72	[58,93]
Decipher	–	–	0.75–0.83	[75,94]
Prolaris (CCR score)	–	–	0.70–0.78	[95,96]
ConfirmMDx	~62–68%	~64–71%	0.70–0.80	[66,97]
